# Aesthetic Medicine Management and the Role of Dermocosmetics for Acne‐Prone Skin: A (Narrative) Mini Review

**DOI:** 10.1111/jocd.70297

**Published:** 2025-07-19

**Authors:** M. Secchi, N. Fraone, I. Ottaviano, S. Floridia, G. Babino, S. Santini, E. Fulgione, E. Bartoletti

**Affiliations:** ^1^ Università Cattolica del Sacro Cuore, International School Carlo Alberto Bartoletti Foundation Rome Italy; ^2^ International School Carlo Alberto Bartoletti Foundation Rome Italy; ^3^ Private Dermatologist Specialist Rome Italy; ^4^ Hospital Medical Director, General Medicine Division, Giovanni Paolo 2 Hospital Ragusa Italy; ^5^ Dermatology Unit Department of Mental and Physical Health and Preventive Medicine, Luigi Vanvitelli University Naples Italy; ^6^ International School Carlo Albero Bartoletti Foundation Rome Italy; ^7^ Dermatologic Clinic Luigi Vanvitelli University Naples Italy; ^8^ Ambulatory Service of Aestethic Medicine for Psychophysical Well‐Being in Pathology Hospital Gemelli Isola Tiberina Rome Italy

**Keywords:** acne, dermocosmetics, monotherapy, patient journey, skin care, skin management

## Abstract

**Background:**

Acne is a prevalent skin condition affecting individuals of various ages, with a peak in the 16–24 age group and a rise in adult acne. It significantly impacts patients' lives, both personally and socially, necessitating a multidisciplinary approach to management. Dermatologists and esthetic doctors play a crucial role in managing acne and acne‐prone skin. The process begins with a thorough consultation involving a comprehensive medical history, clinical and instrumental evaluations to determine the most suitable treatment regimen.

**Aims:**

This article summarizes the latest literature evidence on the esthetic medicine management and dermocosmetics application for acne‐prone skin.

**Methods:**

The authors performed a literature review on selected topics to highlight the role of dermocosmetics, as they come in diverse formulations, featuring single or combined active ingredients with various properties such as sebum control, antimicrobial, anti‐inflammatory, antioxidant, and keratolytic effects.

**Results:**

Dermocosmetics has been categorized as daily‐use products (e.g., moisturizers), short‐term use products (e.g., “SOS” products), cleansers, and sunscreens, and the main evidence on their use has been summarized.

**Conclusions:**

Acne management requires a personalized and multifaceted approach, encompassing lifestyle counseling, pharmacological treatments, and tailored dermocosmetic regimens. By addressing the diverse needs of patients with acne and acne‐prone skin, healthcare professionals can effectively improve their quality of life and well‐being.

## 1 Introduction

Acne and acne‐prone skin represent one of the most common conditions that lead patients to consult medical doctors (MDs) [1, 2].

Acne vulgaris is a chronic inflammatory disorder of the pilosebaceous unit, affecting a significant portion of adolescents and adults, particularly adult women. It manifests as comedones, papules, pustules, nodules, and, in some cases, cysts. Acne‐prone skin is characterized by increased sebum production, follicular hyperkeratinization, microbial colonization (primarily by Cutibacterium acnes), and an inflammatory response [1].

Unlike active acne, acne‐prone skin does not necessarily present with ongoing flare‐ups but is predisposed to developing acne lesions under specific triggers such as hormonal fluctuations, stress, or inappropriate skincare. These features contribute to a predisposition to acne outbreaks and associated skin sensitivity.

Patients with acne often exhibit compromised epidermal barrier function, leading to increased transepidermal water loss (TEWL) and reduced hydration levels. Adult female acne (AFA) is particularly associated with postinflammatory erythema, hyperpigmentation, and increased skin sensitivity. Psychological distress is also commonly reported, making a comprehensive treatment approach necessary.

Acne‐prone skin may also present with signs of inflammation, erythema, hyperpigmentation, and scarring, which negatively impact quality of life (QoL) [1].

Acne is a multifactorial disease involving four primary pathogenic mechanisms: pathophysiology is multifactorial, with increased sebaceous glands and sebum production, hyperkeratinization of the pilosebaceous infundibulum, skin microbiome disbalance, and innate immunity activation [3]:

- excessive sebum production regulated by androgens, leading to sebaceous gland hyperplasia;
- altered follicular hyperkeratinization, which obstructs sebum secretion;
- colonization of the sebaceous gland by Cutibacterium acnes (formerly *Propionibacterium acnes* ), contributing to qualitative sebum alterations that enhance its pro‐inflammatory properties; and
- chronic inflammation driven by complex interactions between innate and adaptive immune mechanisms.

Recent advances in understanding the physiological and molecular mechanisms of acne have highlighted the mechanism of formation of the infra‐clinical earliest lesions in the acne lesional cycle called microcomedones. This process, called the “comedone switch,” results from a loss of homeostasis in sebaceous gland progenitor cells, influenced by factors such as increased sebum free fatty acids, C. acnes, vitamin A deficiency, and xenobiotics. Maintaining homeostasis during sebaceous stem‐cell differentiation is now a key strategy for preventing acne [4].

The related skin lesions and clinical picture can have an important impact on the daily life of patients, also in the esthetic sphere [1].

Management of acne and acne‐prone skin requires trained experts who can provide for adequate overall management, counsel a healthy daily lifestyle, prescribe a tailored dermocosmetic and pharmacological treatment regimen based on subjects' needs, and refer patients to other MDs when needed.

The basic therapeutic strategy for acne care is the control of sebum secretion, abnormal keratinization of follicular epithelium and bacterial infection [5]. Therefore, anti‐inflammatory drugs, bactericidal medications and agents aimed at controlling keratinization are mainly used for acne care.

Nevertheless, beside a personalized therapy, maintaining a well‐balanced skincare routine tailored for acne and acne‐prone skin is crucial to prevent breakouts and minimize irritation. Skincare—including basic routines and use of dermocosmetics—plays a crucial role in managing acne and maintaining skin barrier integrity. Dermocosmetics are skin care products with sophisticated active ingredients that are specifically designed to alleviate symptoms of various skin conditions and preserve or restore a healthy skin barrier physiology. These novel skin care products have undergone rigorous clinical testing, demonstrating their efficacy and safety [5]. The use of dermocosmetic products in acne has acquired a great importance in the last years and can, therefore, enhance treatment efficacy, reduce adverse effects, and improve patient adherence to acne therapies [6]

In the daily skin care, dermocosmetics play an important role in different functions (cleansing, moisturizing, photoprotection) according to the patient journey step (e.g., treatment, maintenance) [5, 7].

Washing the face and body with appropriate cleansers improves acne by removing the hair follicle plugs and preventing the obstruction of hair follicles, while insufficient face washing may exacerbate acne. Cleansers should remove dirt and sebum but maintain lipids and skin moisture. The choice of a cleanser depends on skin type. Patients with oily skin should use a highly rinsable product with no residual moisturizer (such as those based on sodium lauryl sulfate). In patients with other skin types (dry, damaged by the sun or smoke), a noncomedogenic moisturizing liquid cleanser is preferred.

Disruption of the epithelial skin barrier is a relevant feature in acne. However, this dysfunction may also result from aggressive acne treatments, such as over‐the‐counter products or harsh peeling procedures. Clinically, skin barrier impairment presents as dryness and irritation, often experienced as burning or tingling sensations. These symptoms are, at least in part, driven by transepidermal water loss. Consequently, the use of moisturizers plays a crucial role in acne management.

Active ingredients, such as panthenol, ceramides, glycerin, niacinamide, and thermal spring water, contribute to skin barrier restoration. Additionally, keratolytic agents, including alpha hydroxy acids, may support epithelial barrier function by promoting epidermal thickness and regulating hyperkeratinization [6].

Daily management of mild acne and acne‐prone skin with adjunctive dermocosmetics can have a role not only in preventing new acne manifestations but also in supporting the treatment of acne lesions and counteracting some effects of drug therapy to improve patient compliance. Dermocosmetics can maintain skin homeostasis in its multiple aspects (microbiome, skin barrier, sebum) and in the early stages (microcomedones). Different active ingredients were studied for acne and acne‐prone patients, with activities interfering with the pathogenic mechanisms involved in acne: activity on microcomedones, sebum regulators, antimicrobials, anti‐inflammatory agents, keratolytics, and anti‐oxidants. Table 1 summarizes active ingredients with demonstrated activity in acne and acne‐prone patients.

**TABLE 1:** Dermocosmetics for acne management.

Action	Type of products/components	Format
Photoprotection	Filter for both UVB and UVA [5] SPF 30 during winter SPF 50 during summer	Sprays, gels, or liquid (light and nonocclusive products) [5]
Cleansing	Soap free, pH balanced [3] 5 With nonionic and silicone‐based surfactant [5]. May contain alpha hydroxy acids, salicylic acid, benzoyl peroxide, etc. [5]	Cleansing balms, micellar water, milk, gel or oils [5] non‐occlusive products: water–oil emulsions, lotions, gels, creams, serum
Sebum controlling agents	Nicotinamide and Vitamin B3, *Silybum marianum* extract [7] Retinaldehyde [8]	
Antimicrobial agents	Decanediol and tea tree oil [7] Retinaldehyde [9]	
Anti‐inflammatory agents	*Silybum marianum* extract, [7] *Rhealba Oat Plantlet Extract, niacinamide* *Salix alba* , soy isoflavones, enoxolone, *ginkgo biloba* , zinc, panthenol	
Antioxidant agents	Epigallocatechin‐3‐gallate (EGCG), fullerene	
Keratolytics agents	Salicylic acid and retinol derivatives, Bakuchiol [7]	
Skin barrier and microbiome protecting agents	*Silybum marianum* fruit extract [4, 10] Rhealba Oat Plantlet Extract and Myrtacine Ceramides Hyaluronic acid	

Sun exposure does not improve acne but can worsen the appearance of the skin, including sequelae (PIH). In acne patients, it has a big impact on the progression of the disease because it can promote bacterial proliferation and reduce immune response, thus favoring inflammation. Therefore, it is essential to explain the importance of sun protection to the patient. It should be recommended a specific daily photoprotection (also beneficial for other aspects such as prevention of skin cancer and photoaging).

Acute and chronic skin alterations can be triggered not only by ultraviolet radiation (UVR) but also by visible (Vis) and infrared (IR) light. More specifically, high‐energy visible (HEV) light (400–450 nm) belonging to the blue light waveband (400–500 nm) can penetrate from the epidermis to the upper dermis of the skin, where it can have a major effect on skin cells. The use of topical sunscreens containing filters with activities covering a wide range of solar radiation wavelengths is therefore a requisite for effective photoprotection against all forms of solar‐induced damage. Recently, a new filter—TriAsorB^TM^ ‐ was developed by unique optical properties with a protection spectrum that ranges from UVR to HEV/blue light [11].

Cosmetics: make‐up can address the daily needs of patients with acne and provide a quick solution to improve the appearance, enhancing a patient's quality of life, as many other acne products require a sustained period of treatment before results are visible. The bases in cream, powders, or liquid suspensions can act as camouflage. Cosmetics like colored creams and concealers should be oil‐free, noncomedogenic, and should all be removed at night (with special make‐up removers and deep cleansing), as they can still exacerbate acne if left too long. The light foundation is easier to apply on scaly and irritated skin, can be fixed with dust, and is easier to remove than heavy bases [5].

Accordingly, topical dermocosmetic products are often used either as adjunctive therapies to enhance the effectiveness and tolerance of medical anti‐acne therapies and improve treatment adherence, or as monotherapies to prolong remission [3].

This mini review aims to focus and summarize the latest literature evidence on the esthetic medicine management and dermocosmetics application for acne‐prone skin.

## 2 Methods

Since the article structure chosen is a mini review, the authors agreed on a group of topics and selected articles from literature according to the relevance with the relative discussion subject.

The topics of discussion are (a) patient journey and daily life impact, (b) dermocosmetics in monotherapy (subjects not under pharmacological treatment), (c) dermocosmetic support for patients undergoing drug treatment, and (d) dermocosmetic support in case of procedure for acne‐prone skin and sequelae.

Research on Pubmed database has been carried out to find out articles with the following keywords: “acne” AND “dermocosmetics” AND “cosmetics.” The research included literature from 2018, and articles were excluded if not available in English language.

## 3 Results

The research by the “acne” AND “dermocosmetics” keyword accounted for a total of 40 articles, whereas “acne” AND “cosmetics” 655 results. For each topic, the most relevant articles were selected and commented to respect the article scheme and overview aim.

### 3.1 Patient Journey and Daily Life Impact

Nandi and Shrivastava has explored and investigated the so‐called “multifaceted impact” of acne, where multiple factors are involved and a need for an integration of a multidisciplinary approach is required [12]. In literature, a large international survey has pointed out the burden of skin diseases localized on the face region: thus, a special focus on these patients is required to contrast the higher risk of social exclusion and quality of life reduction [13].

Recently, the patient journey and its real‐life management has been investigated in an infodemiologic study: patients often turned to social networks expressing feelings and seeking solutions [14]. Among acne‐related difficulties, therapeutic wandering was frequently mentioned [14]. Here the authors underline the need for training, education, and for fighting against preconceived ideas [14].

### 3.2 Dermocosmetics in Monotherapy (Subjects Not Under Pharmacological Treatment)

Clinical, observational real‐life studies, and reviews have investigated and reported the roles and functions of dermocosmetics alone in acne‐prone skin [5, 7, 15, 16].

Dermocosmetic management for acne‐prone skin have been reviewed in the last years by different authors groups [5, 7, 16]. First, a distinction should be considered according to the function of the overall dermocosmetic formula in the skin care regimen: daily use dermocosmetics (e.g., moisturizers), cleansing products and sunscreens [16]. Moreover, looking at single or combined ingredients, actives functions can be classified as: sebum‐controlling, antimicrobial, anti‐inflammatory, antioxidant and keratolytic [3].

New discoveries in acne pathophysiology have highlighted the homeostasis maintenance around the sebaceous gland progenitor cells as a possible target for skincare products: thus, the new aim in patients with acne‐prone skin could be targeting (and preventing) the microcomedone formation [15].

One of the largest real‐life studies in dermocosmetics is represented by a recent international, multicenter study focused on a dermocosmetic containing a *Silybum marianum* Fruit Extract (SMFE), that has been used as monotherapy in 46.8% of the 4230 patients enrolled [15]. Moreover, this study showed that approximately 95% of all the patients (adolescents and young adults with acne‐prone skin) experienced “good” or “very good” tolerance to this dermocosmetic product containing SMFE, whereas its effectiveness was rated as “effective” or “highly effective” in about 80% of cases. A significant reduction in the mean Global Evaluation of Acne (GEA) grade was observed (−36% ± 39%, *p* < 0.0001) by the end of the study. Additionally, the quality of life (QoL) of most patients (80%) improved, and a majority (79% to 94%) expressed appreciation for the product's cosmetic properties.

This SMFE‐containing product is the first topical formulation identified as capable of modulating the comedone‐switch mechanism we discussed above. In a recent open‐label study, twice‐daily application of SMFE for 1 year in patients with mild‐to‐moderate facial acne demonstrated good tolerability and led to sustained, highly significant reductions in lesion counts, clinical scores, and other efficacy markers over several months. Additionally, the need for any acne medication was recorded on less than 4% of the days during the 12‐month follow‐up period [15].

### 3.3 Dermocosmetic Support for Patients Undergoing Drug Treatment

Many authors and groups have examined the role of dermocosmetics as support to pharmacological therapies [17, 18, 19].

In the last years, an emerging role has been described for dermocosmetics in the management of local side effects related to acne topical therapies such as retinoids or fixed drug combinations [17, 18]. In fact, application of dermocosmetics (moisturizers and cleansers) has been associated with an increase in treatment adherence, thus supporting their role in adjunct to drug treatments [16]. In this context, real‐life experiences have reported the use of dermocosmetics both in association with or after anti‐acne therapy [15].

These data suggest that, when prescribing an anti‐acne drug therapy, the whole picture of the management should be considered, including the mechanism of action and potential related side effects of drugs along with an overall evaluation of skin care needs, from cleansing to photoprotection.

### 3.4 Dermocosmetic Support in Case of Procedure for Acne Prone Skin and Sequelae

Acne sequelae can be targeted by different therapies, including medical procedures such as lasers [16, 17]. Nowadays, the mainstay approach for acne scars considers resurfacing lasers, and an expert panel has recently underlined the need for synergy of different treatment modalities to reach optimal results [16, 17].

## 4 Discussion

According to previous data and a recent global worldwide study, overall acne prevalence has a peak in the 16–24 years old group, but an increase in adult acne is emerging [12]. In fact, acne represents one of the most common dermatological conditions, and its impact on patients' personal and social spheres is relevant [1, 2]. This so‐called “multifaceted impact” requires a multidisciplinary approach in its management, with the integration of multiple trained expert personnel [13].

Patients with acne and acne‐prone skin can present a variety of clinical pictures (noninflammatory and inflammatory lesions, sequelae) and management of these conditions requires trained and qualified experts [5]. Consultation is the first step to choose the best and most suitable treatment regimen for patients with acne and acne‐prone skin: in fact, it provides for counseling a healthy daily lifestyle, prescribing a tailored dermocosmetic and pharmacological treatment regimen, and referring patients to other MDs when needed, after a complete anamnesis, clinical and instrumental evaluations have been carried out [5]. Dermatologists and esthetic doctors have crucial roles in taking care of patients with acne and acne‐prone skin.

In Esthetic Medicine, a defined protocol has been defined and described since the end of the 1970s by Doctor Carlo Alberto Bartoletti and Doctor Gaston Ramette [20]. These authors have worked on a rigorous methodology where all the main elements are collected in a systematic way described by a the so‐called Mediskin check‐up protocol, from which the skin status (“biotype”) and subjects' needs are targeted in an Esthetic Medicine Program [20, 21]. This program includes a dermocosmetic regimen for appropriate and tailored daily skincare [20, 21].

Literature supporting a role for dermocosmetics in acne and acne‐prone skin is broad, and it is increasing year by year. Dermocosmetics play a role in different functions of daily skin care, including cleansing and photoprotection [5, 7] and can be applied in steps of the patients' journey, according to the clinical pictures and subjects' needs (e.g., treatment, maintenance, etc.) [5, 7].

Studies and reviews have analyzed the application of dermocosmetics both in subjects not undergoing pharmacological therapies and in patients requiring antiacne drugs [5, 7]. For example, some patients could benefit from a dermocosmetic therapy in a maintenance phase, whereas a better compliance could be achieved associating cosmetics with antiacne drugs [5, 7].

Each dermocosmetic has its own formula, characterized by single or combined (dermocosmetic) actives that can be classified as: sebum‐controlling, antimicrobial, anti‐inflammatory, antioxidant, and keratolytic [3]. Moreover, their functions allow us to classify them as daily use dermocosmetics (e.g., moisturizers), short‐term use dermocosmetics (e.g., “SOS” products) cleansing products, and sunscreens [5, 7].

Thus, when prescribing a dermocosmetic regimen for patients with acne and acne‐prone skin, esthetic doctors should first evaluate the skin biotype (normal skin; skin with increased content of superficial lipid [up to seborrhea]; dry skin) and the eventually associated pharmacological therapy to then tailor a complete dermocosmetic scheme. The choice of dermocosmetics should consider both the active ingredients and the formula texture: for example, a dry skin treated by anti‐acne drugs (e.g., topical retinoids) could benefit from moisturizers, whereas a seborrheic skin (without pharmacological therapies) could need formulas acting on sebum regulation and microcomedone prevention. Each patient should receive counseling on photoprotection, too, as false myths on photoexposure and acne are still present: thus, the need to recommend a good photoprotection including on UVB, UVA and HEV light that have an impact on skin damage, postinflammatory hyperpigmentation and oxidative stress [22, 23, 24].

To conclude, patients with acne and acne‐prone skin can have an important impact of these conditions on their daily life, and the aim of esthetic doctors is to support them with a tailored program, including counseling on a healthy daily lifestyle and referring patients to other specialists when needed. Moreover, in Esthetic Medicine, a detailed consultation with a defined protocol (the so‐called Mediskin check‐up protocol) can help MDs to have an overall clinical picture and knowledge of the skin status (“biotype”) to prescribe also a tailored dermocosmetic treatment regimen.

By integrating pharmacological and cosmetic treatments, a comprehensive acne management strategy can be achieved, addressing both the pathophysiology and esthetic concerns of acne patients.

## Author Contributions

E.B., M.S., E.F. conceptualization. E.B., M.S., E.F. methodology. All Authors writing review, editing, and approval to submit.

## Conflicts of Interest

The authors declare no conflicts of interest.

## Data Availability

Data sharing is not applicable to this article as no new data were created or analyzed in this study.
